# Meta-Analysis of Direct and Indirect Effects of Father Absence on Menarcheal Timing

**DOI:** 10.3389/fpsyg.2020.01641

**Published:** 2020-07-28

**Authors:** Shaolingyun Guo, Hui Jing Lu, Nan Zhu, Lei Chang

**Affiliations:** ^1^Department of Applied Social Sciences, The Hong Kong Polytechnic University, Kowloon, Hong Kong; ^2^Department of Psychology, University of Macau, Taipa, Macau

**Keywords:** meta-analysis, father absence, childhood stress, menarche, mediation

## Abstract

Despite extensive evidence of the association between father absence and early onset of menarche, whether father absence directly accelerates the onset of menarche or the association is mediated by other negative family psychosocial processes remains unclear. Reliable theories on the basis of which father absence has been investigated also vary. Within the life history (LH) theoretical framework, we conducted a meta-analysis of studies that investigated father absence, menarcheal timing, and various family disturbances that cause stress in children. We tested the hypothesis that father absence exerts a direct effect on menarcheal timing and an indirect effect on menarcheal timing mediated by integrated childhood stress. Quantitative synthesis using a two-stage meta-analytic structural equation modeling approach was applied to test our hypothesis. Based on seven research articles (*N* = 4,619) that include at least one form of family stressor as well as father absence and menarcheal timing, integrated childhood stress emerged as a robust mediator of the association between father absence and early menarcheal timing, and the total effect of father absence on menarcheal timing had reduced in size after accounting for the mediating effect of childhood stress. The findings emphasize the importance of a father figure in regulating a child's LH, including menarcheal timing.

## Introduction

Early childhood experiences deeply influence female reproductive development, including the timing of puberty, which is generally indicated by the age of menarche (Karapanou and Papadimitriou, 2010). Age at menarche depends on the interaction between genes and the environment (DiVall and Radovick, 2008); moreover, the effects of genetic factors do not preclude environmental and psychosocial factors affecting pubertal timing (Graber et al., 1995; Chasiotis et al., 1998; Ellis et al., 1999). To explain the potential effects of environmental factors on menarcheal timing, researchers have proposed different theories, including the paternal investment and resource scarcity hypothesis (Draper and Harpending, 1982; Belsky et al., 1991), male shortage model (Hoier, 2003), and different aspects of the life history (LH) theoretical framework (Draper and Harpending, 1982; Belsky et al., 1991; Chisholm, 1993; Ellis, 2004; Belsky, 2012). However, the explanations as well as the effects of father absence on early onset of menarche reported in these studies are inconsistent or equivocal. Some studies have demonstrated that father absence is associated with early onset of menarche (e.g., Moffitt et al., 1992; Quinlan, 2003; Webster et al., 2014), whereas other studies have shown that father absence did not affect menarcheal timing (Campbell and Udry, 1995; D'Onofrio et al., 2006; Sohn, 2017). In addition, whether father absence directly affects menarcheal timing or the effect is mediated by other mechanisms remains unclear. The present meta-analysis adopted the LH framework to examine empirical research that explored factors accelerating the onset of menarche and investigated the indirect effects of father absence on menarcheal timing.

### Menarcheal Timing and the Life History Strategy

Menarcheal timing is one of the few physical LH traits that have been investigated in human studies (Del Giudice, 2020). Conceptualized within the LH theoretical framework, menarcheal timing assumes to be a good indicator of the fast–slow LH continuum. Early menarcheal timing is associated with the faster end of the continuum that trades growth, development, and learning for early maturation and reproduction. The faster LH strategy results in rapid development, relatively high mating effort, and raising numerous offspring with relatively low parental investment. By contrast, late menarcheal timing is associated with the slow LH strategy, which is characterized by delayed reproduction because of the allocation of bioenergy to somatic development and learning and relatively high parental investment in raising and training high-quality offspring (Ellis et al., 2009; Del Giudice et al., 2015; Ellis and Del Giudice, 2019). The adopted LH trade-off strategy and menarcheal timing vary in response to environmental factors and are determined by environmental constraints. Of the two major environmental constraints that determine animal LH, namely, resource constraint or food shortage and safety constraint or extrinsic risk, food shortage has become less relevant in contemporary human life because of sufficient food supplies that exceed the survival threshold (Chang and Lu, 2018). The diminished role of food shortage over the economic growth is also evident in the stable secular trend of menarcheal age shifting over the past century from the late teenage years to the early teenage years in recent times (Soliman et al., 2014; Chang and Lu, 2018).

The second environmental constraint, namely, safety constraint or extrinsic risk, is particularly relevant in shaping human LH strategies (Ellis et al., 2009). Major safety threats in evolution include predation, disease, and intraspecific violence. These extrinsic risks or safety threats cause mortality and morbidity that are beyond an individual's survival efforts. Known as environmental harshness and unpredictability, the frequency of and variation in extrinsic risks are associated with fast LH strategies (Ellis et al., 2009). In response to environmental harshness and unpredictability, fast strategists who grow rapidly and mature early are more likely to escape death and disability in the post-reproductive period. Moreover, fast strategists who produce many offspring are more likely to hedge against juvenile mortality and achieve higher fitness than individuals who exhibit high parental investment, which is ineffective in preventing juvenile mortality. By contrast, slow LH strategists prevail in safe and predictable environments. They invest time and energy in their own physical and mental development (acquiring knowledge and skills) as well as in the development of their offspring; consequently, generations of slow strategists are more competitive in certain ecological contexts (i.e., where high skills are favored) than fast strategists who allocate higher amounts of energy to mating than to learning and development or parenting and training their offspring.

### Direct and Indirect Effects of Father Absence

As a potential evolved adaptive response, fast–slow LH trade-off strategies continue to regulate development and behavior in response to current environmental constraints (Pepper and Nettle, 2017). According to the literature, contemporary proxies of environmental harshness and unpredictability that have been extensively investigated include low familial socioeconomic status (SES; Belsky et al., 2012), employment and residential changes (Doom et al., 2016; Zuo et al., 2018), unsafe neighborhood and unpredictable life events (Chang et al., 2019), familial chaos (Lu and Chang, 2019), harsh parenting (Mell et al., 2018), and parental absence (Chang and Lu, 2018). Among these microenvironmental proxies, father absence has particularly been implicated in inducing early menarche (Belsky et al., 1991; Ellis, 2004) and other fast LH manifestations (Ellis et al., 2003; Chang and Lu, 2018). Father absence represents environmental harshness and unpredictability because the presence of a father is an essential part of human evolution (Marlowe, 2000; Lu et al., 2015). Paternal care is evidenced in humans by the father assuming the role of a helper at the nest. Paternal and biparental care of the offspring represents one of many interrelated slow LH strategies, including pair bonding, monogamy, and gender equality development (Zhu and Chang, 2019). In terms of both direct parental care and indirect parental investment in the form of provisioning, a father is essential in supporting slow LH strategies and development in human children (Geary, 2000). Father absence causes disruptions in the otherwise stable microenvironment of a child and leads to fast LH manifestations such as early menarche. Concealed ovulation to prolong mate guarding, employment of the father as a helper at the nest, and selecting a suitable mate in terms of paternal value (Lu et al., 2015) are evolutionary events that can also be viewed as advancements of female interest in the long-standing conflict of interests between the sexes. This perspective suggests that father absence should particularly upset daughters' LH and cause early menarche. The aforementioned prediction is consistent with the literature that associates father absence with menarche and girls' LH.

In addition to the direct effect on menarche and other fast LH manifestations, father absence affects child LH indirectly by contributing to familial perturbation and stress, which result in fast LH manifestations including the early onset of menarche (Belsky et al., 1991, 2012). The indirect effect of father absence was proposed within the psychosocial acceleration model that focused on how a harsh family environment accelerates children's reproductive and LH schedules (Belsky et al., 1991). A harsh family environment includes situations such as father absence, marital conflict, divorce or separation, coercive or abusive parenting, and insufficient or inconsistent economic conditions. Any one of these factors may cause perturbations to other components of the family system and create cascades of harsh and stressful experiences for the child. For example, parents experiencing marital difficulties or economic hardships may exhibit insensitivity, rejection, or inconsistency in child-rearing, whereas stable marital and economic conditions are associated with supportive and consistent parenting as well as secure child attachment. Psychosocial stresses resulting from harsh parenting and other family disturbances were reported to accelerate LH including menarcheal timing (Belsky et al., 1991). According to the psychosocial acceleration model (Belsky et al., 1991), father absence represents one example of possible family disturbances, and children experience stress from harsh family environments, which may accelerate LH and cause early onset of menarche.

### Present Study

**Figure 1:**
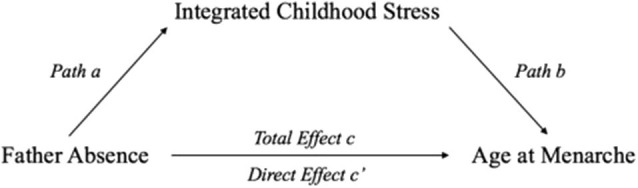
Single mediator model. The total effect is presented as path c, and the direct effect is presented as path c'.

The present study examined the indirect effect of father absence on menarche mediated through integrated childhood stress. A meta-analysis was used to examine statistical robustness of the overall trends by integrating the potential effect across studies. Previous meta-analyses or systematic review only focused on the total effect of father absence (Webster et al., 2014; Sohn, 2017), but these studies did not include other potential variables that may mediate the relation between father absence and menarcheal timing. The present study applied structural equation modeling in a meta-mediation analysis to identify and explicate the process underlying the observed relationship between father absence (independent variable) and menarcheal age (dependent variable) through a mediating process or intervening variable involving stresses experienced by the child (Figure 1). Full mediation refers to when the regression coefficient for path c' is not significantly different from zero; and partial mediation is said to occur if path c' has decreased in size relative to the coefficient for path c but remains significant (Baron and Kenny, 1986). The aforementioned stresses include family conflict, low SES, negative family relationship, and other child-perceived stresses. A two-stage structural equation modeling (TSSEM) approach [see (Cheung and Chan, 2009)] was adopted to identify, examine, and quantify the indirect effect of father absence on menarcheal timing mediated by integrated childhood stress.

## Methods

### Search Strategy and Study Selection

The present meta-analysis included studies obtained after relevant searches in the following electronic databases: PsycINFO, PubMed, and Google Scholar. Relevant publications were systematically searched using their titles, keywords, and abstracts. The keywords used were “menarche” and “stress,” and the related terms used were “childhood stress,” “hardship,” “adversity,” “family conflict,” “violence,” “childhood abuse,” “maltreatment,” “mistreatment,” “low family income,” “financial difficulty,” and “low SES.” Furthermore, the keyword “father absence” and related terms, including “father absence,” “paternal absence,” and “parental absence,” were used. Additional relevant publications were identified by manually searching in reference lists of reviewed articles.

**Figure 2:**
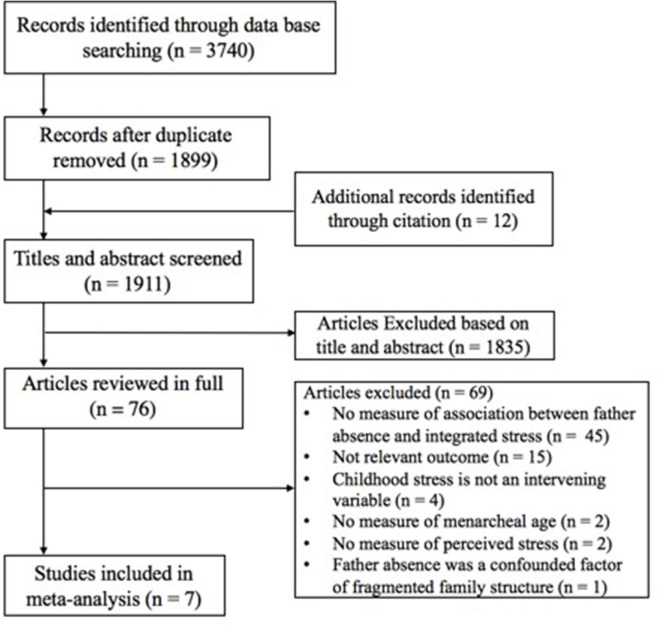
Flow diagram depicting the article selection process.

Studies were selected if they reported (1) the daughters' age or timing of menarche, either concurrently or retrospectively, which was measured using a single item of age or an item embedded in a multi-item measurement of pubertal timing; (2) father absence, missing father figure, or timing and duration of father absence; (3) childhood stress, including perceived childhood or early life stress, stressful family environment, childhood hardship or adversity, early life depression, family conflict, low SES, or childhood abuse; (4) regression coefficients or bivariate correlations between father absence and menarcheal age, between childhood stress and menarcheal age, and between father absence and childhood stress; (5) the mechanism between father absence and menarcheal age through a mediating process or intervening variable involving childhood stresses experienced after father absence. Figure 2 illustrates the flowchart of study selection. Where there were queries regarding the data, authors were contacted for further information; if authors either did not respond or did not provide additional information, we excluded those studies. Table 1 presents the characteristics of the selected studies, including the author(s), year of publication, study sample size, average age or age range of participants, measure of father absence, measure of childhood stress, menarcheal age, and study design of each study.

**Table 1:** Study characteristics.

**References**	**Sample**	***N***	**Age**	**Father absence**	**Types of childhood stress**	**Measures of stressful experiences**	**Puberty timing/menarche**	**Study design**
Chang and Lu (2018)	A community sample was taken from a randomly selected rural county in Henan Province.	57	The average ages of the left-behind and non-left-behind children were 10.78 and 10.69.	Father absence: estimated the amount of time that one or both parents spent away from the child.	Child-perceived stress	12 items from the literature (e.g., Goodman, 1997) to measure this construct (e.g., When I was growing up, they [my parents or people I lived with] fought a lot; they were not around). The items were rated on a 6-point scale.	The age of menarche	Cohort
Culpin et al. (2014)	The sample comprised participants from the Avon Longitudinal Study of Parents and children resident in the former Avon Health Authority in southwest England.	2,750	8 years old up to age 17 years.	A three-level categorical variable to examine potential differential effects of timing of father absence.	Early life socioeconomic stressor	Range of indicators of family socioeconomic disadvantage as major financial problems.	Age at onset of menarche	Cohort
Ellis et al. (1999)	Participating families were initially recruited from three geographical areas (Nashville and Knoxville, Tennessee, and Bloomington, Indiana).	281	Aged 12–13 years old.	Father absence and single-parent status: amount of time the father spent taking care of child, on a 4-point scale ranging from 1 (brief care) to 4 (major care).	Severity of conflict in the parental dyad	Severity of conflict within the parental dyad [this included the biological mother and the male partner (either husband or boyfriend) living in the home] on a 5-point scale ranging from 1 (rarely even shout) to 5 (physical fights, more than once).	Pubertal development scores were converted into pubertal timing scores.	Cohort
James et al. (2012)	Participants were enrolled in a longitudinal study of adolescence in the United States.	240	Mean age = 11.86 years; *SD* = 0.56.	Father absence was reported as 0 = father present; 1 = father absence.	Negative family relationship	Mothers' reports of negative family relationships were assessed *via* Conflict Subscale of the Family Environment Scale (FES), General Functioning Subscale of the Family Assessment Device (FAD), and The Conflict Behavior Questionnaire (CBQ).	Age of menarche	Cohort
Moffitt et al. (1992)	Subjects were adolescent girls involved in the Dunedin (New Zealand) Multidisciplinary Health and Development Study.	326	NA	Mother completed one rating of the amount of time the father spent taking care of child, ranging from 1 (brief care) to 4 (major care).	Family conflict	Mothers were asked to recall each era and to answer this question: “All families have conflicts, parents and kids. What kinds of family strife and violence was your child exposed to during this time” (e.g., shouting, physical fights, pushing—parent–parent or parent–child)?”	Age of menarche	Cohort
Richardson et al. (2018)	Data were collected from a University in the Southeastern United States that serves students from predominantly rural backgrounds.	342	Aged 18–49 years with a mean age of 20.7 years.	Indicators of exposure to the fragmented family structure asking participants to endorse when they experienced before menarche.	Socioeconomic status (SES)	SES was indexed with indicators assessing mother's education, father's education, and childhood family income.	Age at menarche	Cross-sectional
Vigil and Geary (2006)	The participants were women from Albuquerque, New Mexico, and suburban towns in mid-Missouri.	623	Ages 18–56 years, *M* = 26.9 years, *SD* = 8.5.	Father involvement was measured by the amount of time spent with father measured on a 5-point scale, from 1 (never) to 5 (always).	Parent–child conflict	A family conflict item that assessed the degree to which participants argued and fought with their parents during childhood (measured on a 5-point scale).	Age of menarche	Cross-sectional

The validity of each included study was assessed using the STrengthening the Reporting of OBservational studies in Epidemiology (STROBE). All the included studies were evaluated based on the following criteria: the informativeness of the title and abstract, justifiability of the research rationale, rigorousness of the research design, credibility of data sources, accuracy of data analyses, clarity of finding presentation, and relevance of discussion (Vandenbroucke et al., 2014). 14 items adopted from the STROBE were relevant to the aforementioned criteria and assigned a score of 0 or 1. Out of a total score of 14, all the included studies had scores of at least 11 (Table 2), indicating that the validity of the studies based on the STROBE criteria was adequate (Elm et al., 2007; Adams et al., 2018).

**Table 2:** Quality assessments.

**References**	**Chang and Lu (2018)**	**Culpin et al. (2014)**	**Ellis et al. (1999)**	**James et al. (2012)**	**Moffitt et al. (1992)**	**Richardson et al. (2018)**	**Vigil and Geary (2006)**
**Title and abstract**							
Include abstract, an informative and balanced summary.	1	1	1	1	1	1	1
**Introduction**							
Explain the scientific background, objective, and rationale.	1	1	1	1	1	1	1
**Methods**							
Present key elements of study design.	1	1	1	1	1	1	1
Describe the setting, locations, and relevant dates and characteristics of study participants.	1	1	1	1	1	1	1
Give the eligibility criteria and the sources and methods of selection of participants.	0	1	1	1	0	0	0
Clearly define all outcomes, exposures, predictors, potential confounders, and effect modifiers.	1	1	1	1	1	1	1
Give sources of data and details of methods of assessment.	1	1	1	1	1	1	1
Explain how quantitative variables were handled in the analyses.	1	1	1	1	1	1	1
Statistically appropriate/acceptable methods of data analysis used.	1	1	0	1	0	1	0
**Results**							
Give unadjusted estimates and/or confounder-adjusted estimates and their precision.	1	1	1	1	1	1	1
Give a cautious overall interpretation of results.	1	1	1	1	1	1	1
**Discussion**							
Summarize key results with reference to study objectives.	1	1	1	1	1	1	1
Discuss limitations of the study.	1	1	1	1	0	1	1
Discuss the generalizability of the study results.	1	0	1	1	1	1	1

### Data Analysis

A meta-analytic structural equation modeling (SEM; MASEM) approach was used to estimate the mediating effects involving father absence, integrated stress, and menarche onset age. The MASEM approach was combined with SEM and meta-analysis by synthesizing correlations or covariance matrices between X (independent variable), M (mediator), and Y (dependent variable) across studies and fitting SEMs on the pooled correlation or covariance matrix (Landis, 2013). Meta-analyses were performed using the TSSEM approach adopted from the metaSEM package of R (Cheung and Chan, 2005). In the first stage of the TSSEM approach, a pooled correlation matrix was created using the independent variable, mediator, and outcome variable weighted with the sample size of each study (Cheung and Chan, 2009). That is, in order to conduct TSSEM analyses, regression coefficients or bivariate correlations between X (father absence), M (childhood stress), and Y (menarcheal age or menarcheal timing) were extracted from each relevant study. Corresponding study sample sizes were also extracted. If the correlation matrices were not homogeneous, a pooled correlation matrix was estimated and produced (Cheung, 2013). The second stage of the TSSEM approach involved treating the pooled matrix as the observed correlation matrix and fitting a meta-structural mediational model to test the fit of model to the data (see Cheung, 2013). The pooled correlation matrix was used to fit the stage two structural model, with the asymptotic covariance matrix of the pooled correlation matrix as the weight matrix generated by the weighted least squares (WLS) method (Cheung, 2013). As samples, study design and effect sizes were expected to be different across studies; a random-effects model was adopted with the assumption that population correlation matrices may vary. The random-effects model is generally considered to be more conservative in a meta-analysis in the presence of heterogeneity. Unstandardized regression coefficients and their corresponding standard errors extracted from the stage two TSSEM results were then used to conduct a Sobel test to determine the significance of the indirect pathway from father absence on menarcheal age through childhood stress (Sobel, 1982; Preacher and Hayes, 2008). Supplementary R code to this article is outlined in Appendix A.

## Results

### Study Flow and Characteristics

In the database search, 3,740 candidate studies were identified; 1,841 of these studies were duplicates. After additional articles from citation lists were identified, 1,911 studies were obtained. However, after duplicate removal, 1,835 studies were excluded on the basis of titles and abstracts. Thus, 76 studies were available for full-test evaluation. Of these 76 studies, seven included a measure of a childhood stress and sufficient information to calculate bivariate correlations (Figure 2). The characteristics of the seven included articles are shown in Table 1. Among the included seven studies, childhood stress was measured as family or parental conflict (*n* = 3), socioeconomic stress (*n* = 2), negative family relationship (*n* = 1), and perceived childhood stress index (*n* = 1). The study sample size ranged from 57 to 2,750 participants. The proposed mechanisms of childhood stresses are summarized in Table 3.

**Table 3:** Proposed mechanisms of father absence on early menarche through integrated childhood stress.

**Chang and Lu (2018)**	**Culpin et al. (2014)**	**Ellis et al. (1999)**	**James et al. (2012)**	**Moffitt et al. (1992)**	**Richardson et al. (2018)**	**Vigil and Geary (2006)**
Separate measure of paternal absence on pubertal timing (age of menarche for girls), father absence was associated with increased childhood perceived stress (lack of paternal care) that leads to accelerated pubertal timing.	Some of the effects of father absence on age at menarche may be mediated through exposure to financial problems.	Father effects on daughters' pubertal timing should involve more than just father-absent effects; that is, quality of paternal investment and family life stress (greater severity of conflict in the parental dyad) should predict daughters' pubertal timing even within father-present homes.	Within the model: an indirect pathway of father absence—lower quality family relationships—greater age-adjusted pubertal maturation.	Within the model of contextual stress on girls' age at menarche whereby family conflict mediates the relationship between father absence and age of menarche.	Parental resources and status (socioeconomic status is traditionally indexed with indicators of paternal resources) could indirectly affect environmental/family structure effects (e.g., father absence) on age of menarche (see two-part SEM paths).	Investment in social status (financial assistance as proxies for participants' social status) may serve as a cue that mediates the present-day relation between background parental characteristics (father absence) and reproductive development (including the age of menarche).

*Clarifications are given in parentheses*.

### Two-Stage Structural Equation Modeling Results With Mediation Effect

The correlation matrix was extracted and synthesized using data from the seven included studies for the first stage of the TSSEM approach. The value of the Q statistic, which is a measure of the homogeneity of effect sizes, was significant [Q (18) = 144.59, *p* < 0.001], thus indicating that notable variation was present across the selected studies. Because of the large sample size and high heterogeneity across multiple studies, random-effects models were used for analysis in stages 1 and 2 of the TSSEM approach. Correlation matrices were extracted and synthesized from the seven included studies for the first stage of the TSSEM. The total pooled sample size was 4,619. Table 4 presents the pooled correlation coefficients for X (father absence), M (integrated childhood stress), and Y (age at menarche), showing that all three correlations are highly significant.

**Table 4:** Pooled correlation coefficients (*k* = 7) for X (father absence), M (integrated childhood stress), and Y (age at menarche) produced in two-stage structural equation modeling (TSSEM) stage one random-effects model.

	**Father absence**	**Integrated**	**Age at**
		**childhood**	**menarche**
		**stress**	
Father absence	1		
Integrated childhood stress	0.12 (0.04)^***^	1	
Age at menarche	0.13 (0.03)^***^	0.29 (0.05)^***^	1

Standard errors are displayed in parentheses;

*** *p < 0.001*.

**Figure 3:**
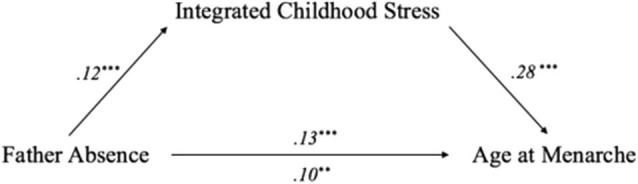
Path analysis diagram depicting two-stage structural equation modeling (TSSEM) stage two random-effects model. The integrated childhood stress partially mediated the causal relationship between father absence and menarcheal age; values are path coefficients. *Notes*: **p < 0.01, p^***^ < 0.001.

A stage 2 analysis using the TSSEM approach was conducted on the pooled correlation matrix as an observed covariance matrix to fit the meta-SEMs by applying the weighted least square estimation method. Figure 3 displays the path diagram of the synthesized meta-SEM results employing TSSEM stage 2 random-effects models. Although the regression coefficient for path c' remained significant, it became reduced in size compared to the value of path c (β = 0.10, *p* = 0.005 for path c'; β = 0.13, *p* < 0.001 for path c), which is an indication of partial mediation (Baron and Kenny, 1986). The Sobel test that used correlation estimates between father absence and childhood stress as well as between childhood stress and menarcheal age and their respective standard errors (SEs) showed that the perceived childhood stress significantly mediated the effects of father absence on menarcheal age (*Z* = 2.64, SE = 0.01, *p* = 0.008). This result is consistent with several key findings reported in the literature on early childhood stress, demonstrating that childhood stress is critical in influencing early menarche (e.g., Belsky et al., 1991; Moffitt et al., 1992; Nettle et al., 2012; Cabeza de Baca and Ellis, 2017).

Generally, the findings supported the role of childhood stress as a mediator, regardless of heterogeneity in measures of childhood stress. Based on the meta-mediation model (Figure 3), father absence was associated with a higher level of childhood stress (β = 0.12, *p* < 0.001), and a higher level of childhood stress was associated with younger menarcheal age (β = 0.28, *p* < 0.001). Although these associations were small to moderate in terms of pooling effect sizes, the findings provide valuable insight into the role of childhood stress in pubertal development. In addition, despite differences among survey questionnaires, study samples, and methods used in the selected studies, findings from all the studies generally demonstrated that early childhood stress was a significant mediator of the effects of father absence on early onset of menarche. The meta-mediation synthesis provided the evidence suggesting that childhood stress was an underlying mechanism that affected the strength of the main association between father absence and menarcheal age in the present study.

## Discussion

The present meta-analysis examined the indirect association between father absence and menarcheal age through the mediation of childhood stress. Compared with extensive evidence that father absence predicts early menarche, no known studies have systematically reviewed and synthesized the mediating effect of childhood stress to account for the father absence effects on early menarche. Previous studies have reported that girls raised in the absence of their fathers are more likely to reach sexual maturation at a younger age (Ellis et al., 2003; Anderson, 2015; Hehman and Salmon, 2018) because the link between father absence and earlier physical maturation is a part of the fast LH strategy (Belsky, 2012; Schlomer et al., 2019). In addition to the total effect of father absence on earlier onset of menarche, the present study showed that father absence and associated stressors, such as familial perturbation and harsh family environment, also affected physical development. The association between menarcheal age and father absence mediated by childhood stress had a smaller regression coefficient than the correlation between father absence and menarcheal age before accounting for the intervening variable. In the seven included studies, integrated childhood stress emerged as a robust mediator, regardless of the heterogeneity among studies and the assumption that all the analyzed studies included random samples from diverse populations.

The present study examined the indirect effect of father absence on the pubertal development of daughters through early life stress. Previous studies have mainly focused on the critical role of father absence in influencing the reproductive strategies of their offspring (Draper and Harpending, 1982; Belsky et al., 1991) or emphasized how menarche was accelerated by childhood stresses, including poor parent–child relationships (Paikoff et al., 1993), children's psychological problems (Moffitt et al., 1992), low SES (Mishra et al., 2009; Belsky et al., 2012), and impoverished and unsafe neighborhoods (Clutterbuck et al., 2014). However, the mechanism through which father absence affects menarcheal age has rarely been examined. Father absence may cause a reduction in household income (because of the lack of material contribution by the father), lack of attention to a child, poor parent–child communication (because the mother has to spend most of her time earning a living), and psychological problems in children because of the harsh survival environment. All consequences of father absence may eventually be internalized into perceived stress by girls, which results in early onset of menarche. This study summarized previous findings and applied meta-mediation analysis to support the aforementioned argument.

The mediating effects of perceived stress in the relationship between father absence and early onset of menarche suggest the possible presence of a causal sequence whereby father absence is a risk factor for the initial perception of familial or psychosocial stress by the child and subsequent early onset of menarche. These findings are consistent with the theoretical underpinnings of stressful circumstances that predict earlier reproductive development (Belsky et al., 1991; Ellis et al., 2003; Tither and Ellis, 2008; Buzney and Decaro, 2012). Performing subgroup analyses of various sources of stress affecting menarcheal timing and longitudinal testing for the aforementioned causal sequence are key directions for future research.

This study has several limitations. First, the present meta-mediation analysis was limited by a relatively small sample size (*n* = 7) because only a limited number of studies have either included the mediation effect or examined the mediation model of father absence or have reported bivariate correlations or regression coefficients between father absence, childhood stress, and menarcheal age. However, the overall effect size of the mediating effects in the included studies was significant according to the results of the Sobel test, suggesting that childhood stress caused by father absence should not be ignored, even if only a few studies have examined the mechanism underlying how father absence affects early onset of menarche. Second, publication bias was possible in the studies used in this meta-analysis, and current findings may overestimate the effects of father absence on accelerated menarcheal age. This may be due to meta-mediation analyses being conducted on *post hoc* basis and thus only being reported when there is evidence of an observed relationship between father absence and age of menarche. Nonetheless, numerous studies have reported the effect of father absence (Draper and Harpending, 1982; Chisholm, 1996; Hoier, 2003; Quinlan, 2003; Jorm et al., 2004) and childhood stress (Belsky et al., 1991; Moffitt et al., 1992; Kim and Smith, 1998; Rodgers and Rowe, 2000) on menarcheal age, suggesting that the published effect was actually observed and reports of the effect were reliable but needed more careful interpretations. In addition, the collapse of different family perturbations (e.g., family or parental conflict, socioeconomic stress, negative family relationship, and perceived childhood stress index) as the child experienced stress may attenuate the expected mediating results because of the heterogeneity of these family perturbations. However, the mediating effect was still significant, suggesting that the mediation of perceived stress in the father absence–menarche relationship was robust. Third, the heterogeneity of pooled effects across the analyzed studies was high in the present meta-analysis. The high heterogeneity can be partially explained by differences in study design, study quality, population characteristics, and variable measurement (Higgins, 2003). Random-effects models in the TSSEM approach were developed and used in the present study to reduce the heterogeneity effects (Cheung and Chan, 2009). Fourth, it should be noted that the lack of a non-WEIRD representative sample may have inhibited the degree of variation in association between father absence and menarcheal age. The mechanism through which father absence affects pubertal timing in developing ecological context may be different, e.g., direct paternal influence may be of more importance in determining reproductive behaviors than timing in non-WEIRD populations (Sheppard et al., 2014) and/or the cause of father absence may differ between WEIRD and non-WEIRD populations where father absence is more likely to be caused by paternal death in non-WEIRD (Sear et al., 2019). As the number and quality of mediation studies increase, it would be worthwhile for future studies to address population-specific mechanisms of integrated childhood stress. Future reviews can reduce heterogeneity by clustering similar stresses into subgroups for further analysis. Despite the aforementioned limitations, this study is the first to holistically analyze published data to examine the indirect effect of father absence on early menarche through stress caused by the lack of the father's role in the child's development.

## Data Availability Statement

The original contributions presented in the study are included in the article/Supplementary Material, further inquiries can be directed to the corresponding author/s.

## Author Contributions

SG, HL, NZ, and LC conceived of the presented idea. SG, HL, and LC designed methods of data collection and analysis. SG performed data analysis and wrote the first draft of the manuscript. HL, NZ, and LC commented on and revised drafts of the paper. All authors discussed the results and contributed to the final version of the paper.

## Conflict of Interest

The authors declare that the research was conducted in the absence of any commercial or financial relationships that could be construed as a potential conflict of interest.
