# Supplementary material for: Meta-Analysis of Direct and Indirect Effects of Father Absence on Menarcheal Timing
Source: Front Psychol. 2020 Jul 28;11:1641. doi: 10.3389/fpsyg.2020.01641 (PMC7399376; doi:10.3389/fpsyg.2020.01641)
Supplement: Supplementary file 1 [file Data_Sheet_1.docx]

Appendix A

library(OpenMx)

## Warning: package 'OpenMx' was built under R version 3.5.2

## To take full advantage of multiple cores, use:
## mxOption(NULL, 'Number of Threads', parallel::detectCores()) #now
## Sys.setenv(OMP_NUM_THREADS=parallel::detectCores()) #before library(OpenMx)

library(metaSEM)

## "SLSQP" is set as the default optimizer in OpenMx.

## mxOption(NULL, "Gradient algorithm") is set at "central".

## mxOption(NULL, "Optimality tolerance") is set at "6.3e-14".

## mxOption(NULL, "Gradient iterations") is set at "2".

library(sem)

##
## Attaching package: 'sem'

## The following object is masked from 'package:OpenMx':
##
## Bollen

library(bmem)

## Loading required package: Amelia

## Loading required package: Rcpp

## ##
## ## Amelia II: Multiple Imputation
## ## (Version 1.7.5, built: 2018-05-07)
## ## Copyright (C) 2005-2020 James Honaker, Gary King and Matthew Blackwell
## ## Refer to http://gking.harvard.edu/amelia/ for more information
## ##

## Loading required package: MASS

## Loading required package: lavaan

## This is lavaan 0.6-3

## lavaan is BETA software! Please report any bugs.

##
## Attaching package: 'lavaan'

## The following objects are masked from 'package:sem':
##
## cfa, sem

## The following object is masked from 'package:OpenMx':
##
## vech

## Loading required package: snowfall

## Loading required package: snow

#Entering the effect size extracted from individual article

x1 <- matrix(c(1,.11,.1,.11,1,.01,.1,.01,1),ncol=3)
dimnames(x1) <- list( c("menarche", "stress", "father absence"), c("menarche", "stress", "father absence") )

x2 <- matrix(c(1,.11,.17,.11,1,.35,.17,.35,1),ncol=3)
dimnames(x2) <- list( c("menarche", "stress", "father absence"), c("menarche", "stress", "father absence") )

x3 <- matrix(c(1,.092,.21,.092,1,0.471,.21,0.471,1),ncol=3)
dimnames(x3) <- list( c("menarche", "stress", "father absence"), c("menarche", "stress", "father absence") )

x4 <- matrix(c(1,0.06,0.15,0.06,1,0.31,0.15,0.31,1),ncol=3)
dimnames(x4) <- list( c("menarche", "stress", "father absence"), c("menarche", "stress", "father absence") )

x5 <- matrix(c(1,0.12,0.18,0.12,1,0.28,0.18,0.28,1),ncol=3)
dimnames(x5) <- list( c("menarche", "stress", "father absence"), c("menarche", "stress", "father absence") )

x6 <- matrix(c(1,0.1,0.11,0.1,1,0.3,0.11,0.3,1),ncol=3)
dimnames(x6) <- list( c("menarche", "stress", "father absence"), c("menarche", "stress", "father absence") )

x7 <- matrix(c(1,0.23,0.04,0.23,1,0.17,0.04,0.17,1),ncol=3)
dimnames(x7) <- list( c("menarche", "stress", "father absence"), c("menarche", "stress", "father absence") )


#Combine each individual effect size

eff <- list(list(x1,x2,x3,x4,x5,x6,x7),c(326,281,2750,623,57,342,240))
names(eff) <- c("data","n")


#Fitting the stage one TSSEM:

random1 <- tssem1(eff$data, eff$n, method="REM",RE.type = "Diag")
summary(random1)

##
## Call:
## meta(y = ES, v = acovR, RE.constraints = Diag(paste0(RE.startvalues,
## "*Tau2_", 1:no.es, "_", 1:no.es)), RE.lbound = RE.lbound,
## I2 = I2, model.name = model.name, suppressWarnings = TRUE,
## silent = silent, run = run)
##
## 95% confidence intervals: z statistic approximation
## Coefficients:
## Estimate Std.Error lbound ubound z value Pr(>|z|)
## Intercept1 0.1188868 0.0359092 0.0485060 0.1892676 3.3108 0.0009304 ***
## Intercept2 0.1337630 0.0342796 0.0665762 0.2009498 3.9021 9.536e-05 ***
## Intercept3 0.2890114 0.0468012 0.1972827 0.3807401 6.1753 6.604e-10 ***
## Tau2_1_1 0.0062945 NA NA NA NA NA
## Tau2_2_2 0.0062952 NA NA NA NA NA
## Tau2_3_3 0.0124736 0.0057591 0.0011860 0.0237612 2.1659 0.0303185 *
## ---
## Signif. codes: 0 '***' 0.001 '**' 0.01 '*' 0.05 '.' 0.1 ' ' 1
##
## Q statistic on the homogeneity of effect sizes: 144.5919
## Degrees of freedom of the Q statistic: 18
## P value of the Q statistic: 0
##
## Heterogeneity indices (based on the estimated Tau2):
## Estimate
## Intercept1: I2 (Q statistic) 0.7510
## Intercept2: I2 (Q statistic) 0.7589
## Intercept3: I2 (Q statistic) 0.8887
##
## Number of studies (or clusters): 7
## Number of observed statistics: 21
## Number of estimated parameters: 6
## Degrees of freedom: 15
## -2 log likelihood: -41.65077
## OpenMx status1: 0 ("0" or "1": The optimization is considered fine.
## Other values may indicate problems.)

##correlation matrix
coef(random1)

## Intercept1 Intercept2 Intercept3 Tau2_1_1 Tau2_2_2 Tau2_3_3
## 0.118886810 0.133762985 0.289011420 0.006294478 0.006295236 0.012473587

#Fitting the stage two TSSEM:


## Prepare a model implied matrix
## Factor correlation matrix

## Create Amatrix
A1 <- create.mxMatrix(c(0,0,0,
 "0.3*x2m",0,0,
 "0.3*x2y","0.3*m2y",0),type="Full",byrow=TRUE,ncol=3,nrow=3,as.mxMatrix=FALSE)
dimnames(A1)[[1]] <- dimnames(A1)[[2]] <- c("X","M","Y")


## Create Smatrix
S1 <- create.mxMatrix(c(1,0,0,
 0,1,0,
 0,0,1), byrow=TRUE,type="Full",ncol=3,nrow=3,as.mxMatrix=FALSE)
dimnames(S1)[[1]] <- dimnames(S1)[[2]] <- c("X","M","Y")


## Create Fmatrix
## CREATES "F MATRIX" WHICH SPECIFIES A SELECTION MATRIX USED TO OBSERVED VARIABLES
F1 <- create.Fmatrix(c(1, 1, 1), name = "F1")


#Stage two TSSEM:
random2 <- tssem2(random1, Amatrix = A1, Smatrix = S1, Fmatrix = F1, diag.constraint = FALSE)

summary(random2)

##
## Call:
## wls(Cov = pooledS, aCov = aCov, n = tssem1.obj$total.n, Amatrix = Amatrix,
## Smatrix = Smatrix, Fmatrix = Fmatrix, diag.constraints = diag.constraints,
## cor.analysis = cor.analysis, intervals.type = intervals.type,
## mx.algebras = mx.algebras, model.name = model.name, suppressWarnings = suppressWarnings,
## silent = silent, run = run)
##
## 95% confidence intervals: z statistic approximation
## Coefficients:
## Estimate Std.Error lbound ubound z value Pr(>|z|)
## x2m 0.118887 0.035909 0.048506 0.189268 3.3108 0.0009304 ***
## m2y 0.277024 0.047621 0.183689 0.370359 5.8173 5.98e-09 ***
## x2y 0.100828 0.035652 0.030951 0.170705 2.8281 0.0046823 **
## ---
## Signif. codes: 0 '***' 0.001 '**' 0.01 '*' 0.05 '.' 0.1 ' ' 1
##
## Goodness-of-fit indices:
## Value
## Sample size 4619.00
## Chi-square of target model 0.00
## DF of target model 0.00
## p value of target model 0.00
## Number of constraints imposed on "Smatrix" 0.00
## DF manually adjusted 0.00
## Chi-square of independence model 60.63
## DF of independence model 3.00
## RMSEA 0.00
## RMSEA lower 95% CI 0.00
## RMSEA upper 95% CI 0.00
## SRMR 0.00
## TLI -Inf
## CFI 1.00
## AIC 0.00
## BIC 0.00
## OpenMx status1: 0 ("0" or "1": The optimization is considered fine.
## Other values indicate problems.)

#Extract the correlation matrix:
vec2symMat(coef(random2, select="fixed"), diag=FALSE)

## [,1] [,2] [,3]
## [1,] 1.0000000 0.1188868 0.2770242
## [2,] 0.1188868 1.0000000 0.1008285
## [3,] 0.2770242 0.1008285 1.0000000
